# Effect of postoperative goal-directed therapy in cancer patients undergoing high-risk surgery: a randomized clinical trial and meta-analysis

**DOI:** 10.1186/s13054-018-2055-4

**Published:** 2018-05-23

**Authors:** Aline Rejane Muller Gerent, Juliano Pinheiro Almeida, Evgeny Fominskiy, Giovanni Landoni, Gisele Queiroz de Oliveira, Stephanie Itala Rizk, Julia Tizue Fukushima, Claudia Marques Simoes, Ulysses Ribeiro, Clarice Lee Park, Rosana Ely Nakamura, Rafael Alves Franco, Patricia Inês Cândido, Cintia Rosa Tavares, Ligia Camara, Graziela dos Santos Rocha Ferreira, Elisangela Pinto Marinho de Almeida, Roberto Kalil Filho, Filomena Regina Barbosa Gomes Galas, Ludhmila Abrahão Hajjar

**Affiliations:** 1 Intensive Care Unit and Department of Anesthesiology, Instituto do Cancer, Hospital das Clinicas da Faculdade de Medicina da Universidade de Sao Paulo, Sao Paulo, Brazil; 2 Department of Anesthesia and Intensive Care, IRCCS San Raffaele Scientific Institute, Milan, Italy; 3 Department of Surgery, Instituto do Cancer, Hospital das Clinicas da Faculdade de Medicina da Universidade de Sao Paulo, Sao Paulo, Brazil; 4 Department of Cardiopneumology, Instituto do Coracao, Hospital das Clinicas, Faculdade de Medicina da Universidade de Sao Paulo, Sao Paulo, Brazil

**Keywords:** Goal-directed therapy, Cancer, High-risk surgery, Mortality, Randomized clinical trial, Meta-analysis

## Abstract

**Background:**

Perioperative goal-directed hemodynamic therapy (GDHT) has been advocated in high-risk patients undergoing noncardiac surgery to reduce postoperative morbidity and mortality. We hypothesized that using cardiac index (CI)-guided GDHT in the postoperative period for patients undergoing high-risk surgery for cancer treatment would reduce 30-day mortality and postoperative complications.

**Methods:**

A randomized, parallel-group, superiority trial was performed in a tertiary oncology hospital. All adult patients undergoing high-risk cancer surgery who required intensive care unit admission were randomly allocated to a CI-guided GDHT group or to a usual care group. In the GDHT group, postoperative therapy aimed at CI ≥ 2.5 L/min/m^2^ using fluids, inotropes and red blood cells during the first 8 postoperative hours. The primary outcome was a composite endpoint of 30-day all-cause mortality and severe postoperative complications during the hospital stay. A meta-analysis was also conducted including all randomized trials of postoperative GDHT published from 1966 to May 2017.

**Results:**

A total of 128 patients (64 in each group) were randomized. The primary outcome occurred in 34 patients of the GDHT group and in 28 patients of the usual care group (53.1% vs 43.8%, absolute difference 9.4 (95% CI, − 7.8 to 25.8); *p* = 0.3). During the 8-h intervention period more patients in the GDHT group received dobutamine when compared to the usual care group (55% vs 16%, *p* < 0.001). A meta-analysis of nine randomized trials showed no differences in postoperative mortality (risk ratio 0.85, 95% CI 0.59–1.23; *p* = 0.4; *p* for heterogeneity = 0.7; *I*^2^ = 0%) and in the overall complications rate (risk ratio 0.88, 95% CI 0.71–1.08; *p* = 0.2; *p* for heterogeneity = 0.07; *I*^2^ = 48%), but a reduced hospital length of stay in the GDHT group (mean difference (MD) – 1.6; 95% CI – 2.75 to − 0.46; *p* = 0.006; *p* for heterogeneity = 0.002; *I*^2^ = 74%).

**Conclusions:**

CI-guided hemodynamic therapy in the first 8 postoperative hours does not reduce 30-day mortality and severe complications during hospital stay when compared to usual care in cancer patients undergoing high-risk surgery.

**Trial registration:**

www.clinicaltrials.gov, NCT01946269. Registered on 16 September 2013.

**Electronic supplementary material:**

The online version of this article (10.1186/s13054-018-2055-4) contains supplementary material, which is available to authorized users.

## Background

For many patients with solid tumors, surgery remains the mainstay of therapy. For these patients, a complication-free operative procedure is vital to maximize the chances that oncological treatment is successful. Postoperative care of cancer patients having major abdominal surgery is challenging because of the unusually long duration of the surgical procedures, the significant fluid and blood losses that can occur, the inherent immunological disturbs and the increased operative risk, as demonstrated by previous studies [1, 2].

Goal-directed hemodynamic therapy (GDHT) comprising rational use of fluids, inotropes, vasopressors and red blood cell (RBC) transfusion according to hemodynamic targets to improve oxygen delivery (DO_2_) can provide outcome benefits in high-risk patients [3]. Even if some studies suggested that GDHT in high-risk patients undergoing surgery was associated with a significant reduction in morbidity and mortality [4–6], recent evidence suggests that the benefits are less than previously hypothesized because of the potential harm of fluid overload, drugs side effects and invasive monitoring [7, 8].

In addition, in most studies GDHT was investigated during surgery and extending for the first 6–12 h after intensive care unit (ICU) admission. It is unclear whether the potential benefits of GDHT are present when GDHT is used during the whole perioperative period or if GDHT use only in the postoperative ICU setting might result in clinical benefits or even in harmful interventions.

Therefore, we performed a randomized clinical trial (RCT) to evaluate the effects of a postoperative GDHT protocol on a composite endpoint of 30-day all-cause mortality and severe postoperative complications during the hospital stay in high-risk cancer patients undergoing surgery. We also conducted an updated systematic review focusing on postoperative GDHT and incorporating the findings of this trial.

## Methods

The Goal-Directed Resuscitation in Cancer Surgery (GRICS II) trial was a single-center, parallel and randomized trial performed at the Cancer Institute, University of Sao Paulo, Sao Paulo, Brazil. The study was approved by the Faculty of Medicine Ethics Committee (number 335/13). This trial was registered at ClinicalTrials.gov on September 16, 2013 (NCT01946269). Written informed consent was obtained from all subjects or their legal surrogates prior to enrolment in the study. Patients were enrolled from October 2013 to September 2015. The trial was overseen by an independent data and safety monitoring board. The two funding sources were the University of Sao Paulo (Brazil) and Edwards LTDA (Irvine, CA, USA), which had no other role in the study. All the authors vouch for the fidelity of the study to the trial protocol and for the accuracy of the data and data analyses.

### Participants

Consecutive patients who were 18 years old or more and scheduled for cancer surgery at the Cancer Institute were screened for enrolment. Patients undergoing high-risk surgery for cancer treatment (upper and lower gastrointestinal tract, urogenital tract, liver or biliary tract surgeries) with duration longer than 90 min and requiring ICU admission were included in the study. Patients were excluded from the study if one of the following was present: declined consent; emergency surgery; enrolled in another study; weight under 55 kg or over 140 kg; active bleeding; contraindication to invasive hemodynamic monitoring; hemodynamic instability (norepinephrine higher than 1 μg/kg/min); surgery for palliative treatment only; or presence of cardiac arrhythmia (atrial fibrillation, frequent ectopy or other dysrhythmias).

### Randomization

Randomization was performed, with a computer-generated list in a 1:1 ratio generated online by a web-based program that ensured allocation concealment.

The investigator opened the serially numbered, sealed, opaque envelopes on arrival of the patients at the ICU, provided exclusion criteria were not met. The nature of the intervention precluded blinding of the patients and attending physicians. Outcome assessors were unaware of the assigned treatment.

The decision to admit the patients to the ICU was made by clinical staff and registered before randomization and surgery.

### Study treatments

#### All patients

Anesthetic management and perioperative care of patients were standardized and are described in detail in Additional file 1: Appendix 1. Briefly, all patients were monitored with a central venous line and indwelling radial artery catheter. Fluids, vasopressor and inotropic agents were administered to maintain mean arterial pressure (MAP) ≥ 65 mmHg, urinary output > 0.5 ml/kg/h, oxygen venous saturation ≥ 70% and lactate levels < 3 mmol/L. Preload was optimized by fluid loading until pressure pulse variation (PPV) was < 10%. Interventions included fluid resuscitation with lactated Ringer’s preferentially, administration of dobutamine and RBC transfusion if needed according to hemodynamic data.

After ICU admission, all patients received care to keep adequate temperature (36 °C), oxygen saturation ≥ 95%, MAP ≥ 65 mmHg, heart rate (HR) between 70 and 100 bpm, central venous oxygen saturation (ScvO_2_) > 70%, lactate level < 3 mmol/L, urinary output > 0.5 ml/kg/h and normoglycemia (140–180 mmol/L). Additional fluids and vasoactive drugs could be administered by the physicians guided by HR, MAP, urine output, venous central oxygen saturation, serum lactate and base excess. Analgesia was provided in most cases by epidural infusion. In a few cases when an epidural technique was not possible, intravenous infusion of morphine was performed.

#### GHDT group

A cardiac output monitor - Vigileo - Edwards LTDA (Irvine, CA, USA) was connected to the radial artery and the cardiac index (CI) and stroke volume index (SVI) values were used as goals to deliver the hemodynamic intervention.

The protocol started at ICU admission and lasted 8 h. The main goal was to achieve CI ≥ 2.5 L/min/m^2^. Initially, fluid resuscitation was provided with the administration of an intravenous bolus in 15 min of 200 ml of Ringer lactate solution with 50 ml of 20% albumin solution if the SVI was less than 35 ml/m^2^. Fluid administration was repeated accordingly to responsiveness and interrupted if SVI ≥ 35 ml/m^2^. The definition of positive response to fluid challenge was an increase in cardiac index ≥ 15% from baseline.

**Fig. 1:**
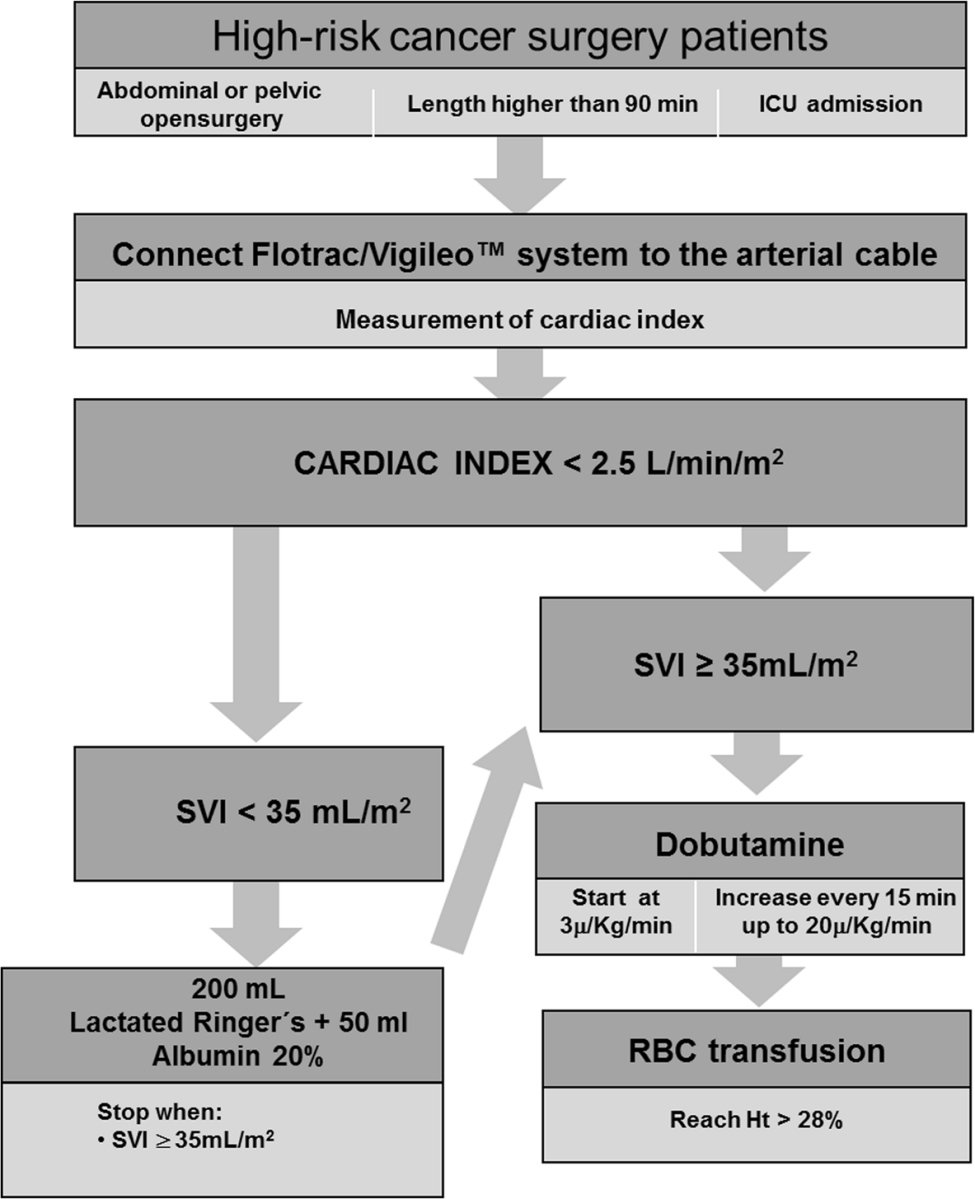
Algorithm of treatment in the goal-directed hemodynamic therapy (GDHT) group. *ICU* intensive care unit, *SVI* stroke volume index, *RBC* red blood cell, *Ht* hematocrit

If the CI remained < 2.5 L/min/m^2^, dobutamine infusion was started at 3 μg/kg/min and titrated every 15 min up to a maximum dose of 20 μg/kg/min. If these interventions did not result in CI ≥ 2.5 L/min/m^2^, RBCs were transfused 1 unit at a time to achieve hematocrit > 24% (Fig. 1).

#### Usual care group

For patients in the usual care group, the institutional protocol was followed to achieve HR between 70 and 100 bpm, ScvO_2_ > 70%, lactate level < 3 mmol/L, hematocrit value > 28%, MAP ≥ 65 mmHg and urinary output > 0.5 ml/kg/h through fluid resuscitation with Ringer lactate solution, administration of dobutamine and RBC transfusion.

### Study outcomes

The primary endpoint was a composite endpoint of 30-day mortality and major clinical complications (Additional file 1: Appendix 2) during the hospital stay. Clinical complications were acute kidney injury (AKI), stroke, myocardial infarction, acute decompensated heart failure, pulmonary thromboembolism, mesenteric ischemia, peripheral vascular ischemia, acute respiratory distress syndrome (ARDS), deep wound infection and reoperation.

Secondary endpoints included the incidence of septic shock, development of AKI requiring renal replacement therapy (RRT), ICU readmission rate, ICU and hospital lengths of stay (LOS), 7-day sepsis-related organ failure assessment (SOFA) score and 90-day mortality (Additional file 1: Appendix 2).

We also analyzed the following hemodynamic parameters and perfusion indexes during the intervention period (at 0 h, 2 h, and 8 h): HR, MAP, ScvO_2_, lactate levels, base excess and CO_2_ gap.

### Data collection and definition of complications

Data were collected by two blinded assessors with more than 3 years of work experience in the cancer ICU. Patients were discharged from the ICU if their physiologic status was stable, there was no need for monitoring and active interventions were not planned. Follow-up after hospital discharge was performed by telephone on the 30th postoperative day (Additional file 1: Appendix 3).

### Systematic review

We also conducted a meta-analysis of all published RCTs following the PRISMA guidelines and comparing postoperative GDHT with standard therapy in noncardiac surgery adult high-risk patients. The PubMed/Medline, Embase and Scopus databases were searched from inception up to May 1, 2017. Details on the PubMed search strategy are provided in Additional file 1: Appendix 4. Two researchers independently screened the titles and abstracts of all initially identified studies according to the selection criteria and extracted the required data. Consensus was reached in the case of any inconsistency with involvement of a third author. We included only studies reporting mortality data (primary outcome of the meta-analysis). Secondary endpoints were overall complications rate and hospital length of stay. Detailed methods are described in Additional file 1: Appendix 4.

### Statistical analysis

Considering a two-sided alpha level of 0.05 and a statistical power of 90%, we calculated that a sample size of 128 patients would be required to reduce the incidence of the composite endpoint from 56% in the control group to 28% in the GDHT group [5]. All analyses were conducted according to the intention-to-treat principle. No assumptions were made for missing or unavailable data. We report continuous variables as mean with standard deviation (SD) or median with interquartile range (IQR), and categorical variables as proportions. Continuous variables were compared using a Student’s *t* test or Mann–Whitney *U* test and categorical variables using Pearson χ^2^ or Fisher exact or likelihood ratio test. Comparisons of SOFA score over time were made using nonparametric Friedman test. Kaplan–Meier curves were built for event-free survival probability up to 30 days following surgery. Two-sided *p* < 0.05 was considered statistically significant.

For the meta-analysis, treatment effects were reported as risk ratio (RR) with 95% confidence interval (CI) for mortality and complications or mean differences with standard deviations for hospital length of stay. The Mantel–Haenszel method was used to combine summary measures using a random-effects model. We evaluated heterogeneity between studies using Cochran’s *Q* and the *I*^2^ statistic. We assessed the potential of publication bias through funnel plot generation and Egger’s regression test.

SPSS version 18.0 (SPSS Inc., Chicago, IL, USA), Review Manager (RevMan, Version 5.3, 2014; The Nordic Cochrane Centre, The Cochrane Collaboration, Copenhagen, Denmark) and STATA (Version 11; StataCorp, College Station, TX, USA) were used for all statistical analyses.

## Results

### Study population

**Fig. 2:**
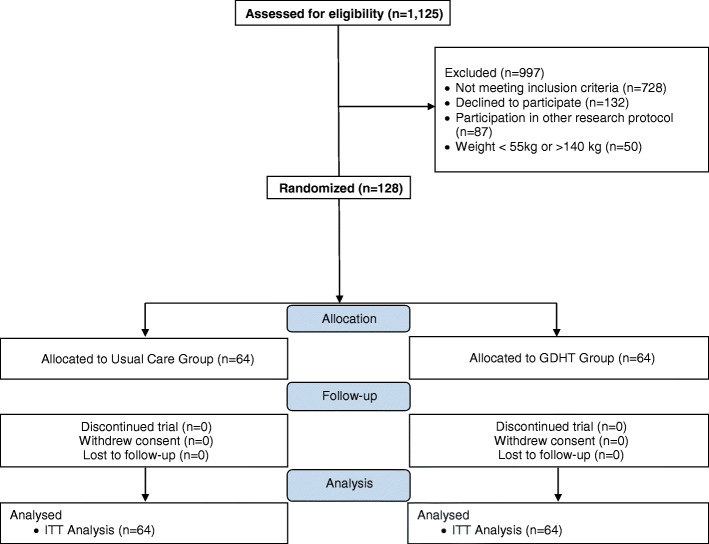
Study flow chart. *GDHT* goal-directed hemodynamic therapy, *ITT* intention-to-treat

One hundred and twenty-eight patients were included in the study (Fig. 2). All patients had outcome data collected and completed the follow-up. Patients were 67 years old and had a good performance status prior to surgery (Table 1), the most commonly performed being gastrointestinal followed by liver and biliary tract surgery. The groups were similar regarding the intraoperative amount of fluids, vasoactive drugs and RBC transfusion received (Table 2).

**Table 1:** Baseline and demographic patients’ characteristics

Variable	GDHT group (*n* = 64)	Usual care group (*n* = 64)
Age (years), mean ± SD	66 ± 12	68 ± 11
BMI (kg/m^2^), median (IQR)	25 (21–31)	25 (21–30)
Sex (male), *n* (%)	32 (50%)	37 (58%)
Race (white), *n* (%)	48 (75%)	52 (81%)
Charlson score^a^, median (IQR)	5 (4–6)	6 (5–8)
ECOG score^b^, *n* (%)		
0	29 (47%)	26 (43%)
1	19 (31%)	19 (32%)
2	6 (10%)	8 (13%)
3	8 (13%)	6 (10%)
4	0	1 (2%)
Karnofsky score^c^, median (IQR)	90 (80–100)	90 (80–100)
Neoplasm, *n* (%)		
Stomach	0	1 (2%)
Esophagus	9 (13%)	6 (10%)
Colon–rectus	8 (13%)	6 (10%)
Gynecological	18 (30%)	21 (34%)
Pancreas	9 (15%)	9 (15%)
Liver	6 (10%)	2 (3%)
Prostate	6 (10%)	8 (13%)
Kidney	4 (7%)	5 (8%)
Others	1 (2%)	3 (5%)
Recent chemotherapy, *n* (%)	3 (5%)	1 (2%)
Recent radiotherapy, *n* (%)	0	0
Smokers, *n* (%)		
No	41 (64%)	41 (64%)
Yes	9 (14%)	7 (11%)
Previously	14 (22%)	16 (25%)
Alcoholism, *n* (%)		
No	56 (88%)	55 (86%)
Yes	4 (6%)	2 (33%)
Previously	4 (6%)	7 (11%)
Hypertension, *n* (%)	38 (59%)	35 (55%)
Diabetes mellitus, *n* (%)	12 (19%)	16 (25%)
Heart failure, *n* (%)	2 (3%)	3 (5%)
Chronic obstructive pulmonary disease, *n* (%)	4 (6%)	6 (9%)
Chronic kidney disease, *n* (%)	2 (3%)	6 (9%)
Hypothyroidism, *n* (%)	5 (8%)	4 (6%)
Stroke, *n* (%)	0	6 (9%)
Acute myocardial infarction, *n* (%)	4 (6%)	5 (8%)
Pulmonary embolism/deep vein thrombosis, *n* (%)	0	1 (2%)

*GDHT* goal directed hemodynamic therapy, *SD* standard deviation, *BMI* body mass index, *IQR* interquartile range, *ECOG* Eastern Cooperative Oncology Group

^a^Comorbidity score

^b^Status performance in cancer patients

^c^Performance status scale

**Table 2:** Surgical-related characteristics

Variable	GDHT group (*n* = 64)	Usual care group (*n* = 64)
Surgery type, *n* (%)		
Upper gastrointestinal tract	21 (33%)	13 (20%)
Lower gastrointestinal tract	15 (23%)	14 (22%)
Liver and biliary tract	2 (3%)	5 (8%)
Urogenital tract	15 (23%)	22(34%)
Others	11 (17%)	10 (16%)
Duration of surgery (min), median (IQR)	360 (273–480)	300 (240–435)
Fluids (ml), median (IQR)	4000 (3000–5763)	4000 (3000–5225)
Norepinephrine, *n* (%)	20 (31%)	19 (30%)
Dobutamine, *n* (%)	0	4 (6%)
Vasopressin, *n* (%)	1 (2%)	0
RBC transfusion, *n* (%)	19 (30%)	15 (23%)
Fresh frozen plasma, *n* (%)	2 (3%)	2 (3%)
Cryoprecipitate, *n* (%)	0	1 (2%)
Platelets, *n* (%)	1 (2%)	0

*GDHT* goal directed hemodynamic therapy, *IQR* interquartile range, *RBC* red blood cell

### Intervention period

In the first 8 h after ICU admission, there was no difference between groups in the volume of administered fluids (1195 ± 719 ml GDHT group vs 1290 ± 609 ml usual care group, *p* = 0.4) and in the RBC transfusion rate (3.1% GDHT group vs 0% usual care group, *p* = 0.5). There was a higher use of dobutamine in the GDHT group when compared to the usual care group (35 patients (55%) vs 10 patients (15%), *p* < 0.001). The CI of the GDHT group patients during the 8-h intervention is reported in Additional file 1: Figure S1. There were no differences between groups in the hemodynamic parameters or perfusion indexes (HR, MAP, ScvO_2_, lactate levels, base excess and PCO_2_ gap; Table 3).

**Table 3:** Hemodynamic variables and tissue perfusion markers during 8-h intervention period

Variable	GDHT group (*n* = 64)	Usual care group (n = 64)	*p*
ΔPCO_2_ (%)			0.9
0 h	6.8 ± 4.4	6.8 ± 4.9	
2 h	7.5 ± 5.8	6.9 ± 4.7	
8 h	6.0 ± 3.3	6.8 ± 4.2	
BE (mmol)			0.9
0 h	−3.7 ± 2.9	−3.7 ± 3.0	
2 h	−4.1 ± 3.7	−3.4 ± 2.9	
8 h	−2.4 ± 2.7	−2.9 ± 3.0	
Lactate (mmol/L)			0.9
0 h	2.6 ± 1.5	2.8 ± 2.3	
2 h	2.9 ± 2.0	3.0 ± 2.3	
8 h	2.9 ± 1.8	3.0 ± 2.5	
HR (bpm)			0.9
0 h	83.1 ± 21.6	85.5 ± 23.9	
2 h	82.9 ± 20.7	83.9 ± 22.6	
8 h	88.2 ± 21.3	82.9 ± 18.9	
MAP (mmHg)			0.3
0 h	94.5 ± 19.6	91.6 ± 17.8	
2 h	88.1 ± 15.6	87.3 ± 14.7	
8 h	85.3 ± 16.9	82.1 ± 15.2	
ScvO_2_ (%)			0.2
0 h	75.7 ± 9.0	77.1 ± 9.0	
2 h	75.6 ± 8.7	77.4 ± 6.6	
8 h	78.7 ± 6.6	76.4 ± 8.0	
CI (L/min/m^2^)			
0 h	2.9 ± 1.0	–	
2 h	2.9 ± 0.9	–	
8 h	3.1 ± 1.0	–	

Data expressed as mean ± standard deviation

*GDHT* goal-directed hemodynamic therapy, *ΔPCO*_*2*_ dioxide carbon gap, *BE* base excess, *HR* heart rate, *MAP* mean arterial pressure, *ScvO*_*2*_ central venous oxygen saturation, *CI* cardiac index

### Primary outcome

The primary composite endpoint of 30-day all-cause mortality and severe clinical complications occurred in 34 patients (53%) in the GDHT group and in 28 patients (44%) in the usual care group (*p* = 0.3). No significant differences were observed between the GDHT and usual care groups in the rate of mortality (14% vs 9%, *p* = 0.4) and of the other severe complications (Table 4), with AKI (51% vs 39%, *p* = 0.184) and reoperation (8% vs 5%, *p* = 0.7) being the most frequently represented.

**Table 4:** Primary outcome

Variable	GDHT group	Usual care group	Absolute difference (95% CI)	*p*
	(*n* = 64)	(*n* = 64)		
Composite endpoint	53 (40–66)	44 (31–57)	9.4 (−7.8 to 25.8)	0.3
Mortality	14 (7–25)	9 (4–19)	4.7 (−6.9 to 16.4)	0.4
Stroke	0 (0–6)	2 (0–8)	−1.6 (−8.3 to 4.2)	0.9
AKI	51 (36–61)	39 (27–52)	9.4 (− 7.6 to 25.6)	0.2
ARDS	5 (2–15)	2 (0–8)	4.7 (−3.1 to 13.5)	0.4
Acute myocardial infarction	2 (0–8)	5 (0.9–13)	−3.1 (− 11.4 to 4.3)	0.6
Acute heart failure	0 (0–6)	2 (0–8)	−1.6 (− 8.3 to 4.2)	0.5
Mesenteric ischemia	2 (0–8)	0 (0–6)	1.6 (−4.2 to 8.3)	0.9
Peripheral vascular ischemia	0 (0–6)	3 (0.3–11)	− 3.1 (− 10.7 to 3.0)	0.5
Pulmonary embolism	0 (0–6)	0 (0–6)	–	–
Deep wound infection	3 (0.3–11)	8 (3–17)	−4.7 (− 14.2 to 4.1)	0.4
Reoperation	8 (3–17)	5 (0.9–13)	3.1 (− 6.2 to 12.8)	0.7

Data presented as % (95% CI)

*GDT* goal-directed hemodynamic therapy, *CI* confidence interval, *AKI* acute kidney injury, *ARDS* acute respiratory distress syndrome

### Secondary outcomes

We did not observe significant differences in any of the secondary outcomes between the GDHT and usual care groups: septic shock (16% vs 13%, *p* = 0.6), AKI needing RRT (13% vs 8%, *p* = 0.4), ICU readmission (13% vs 9%, *p* = 0.6), ICU stay (3 (2–8) days vs 3 (2–5) days, *p* = 0.6) and hospital LOS (11 (6–19) days vs 10 (6–15) days, *p* = 0.4; Table 5).

**Table 5:** Secondary outcomes

Variable	GDHT group (*n* = 64)	Usual care group (*n* = 64)	Absolute difference (95% CI)	*p*
Septic shock	16 (8–27)	13 (6–23)	3.1 (−9.3 to 15.5)	0.611
AKI AKIN 3 and RRT	13 (6–23)	8 (3–17)	4.7 (− 6.3 to 15.9)	0.380
ICU readmission	13 (6–23)	9 (4–19)	3.1 (− 8.2 to 14.6)	0.571
Length of ICU stay (days), median (IQR)	3 (2–8)	3 (2–5)	–	0.571
Length of hospital stay (days), median (IQR)	11 (6–19)	10 (6–15)	–	0.354
SOFA score, mean ± SD				0.308
Admission	4.3 ± 2.4	3.6 ± 2.2	−0.7 (− 1.5 to 0.1)	
D1	4.0 ± 2.5	3.2 ± 2.3	− 0.8 (− 1.6 to 0)	
D2	2.8 ± 2.8	2.1 ± 2.2	− 0.7 (− 1.6 to 0.2)	

Data presented as % (95% CI)

*GDT* goal-directed hemodynamic therapy, *CI* confidence interval, *AKI* acute kidney injury, *AKIN* Acute Kidney Injury Network, *RRT* renal replacement therapy, *ICU* intensive care unit, *IQR* interquartile range, *SOFA* Sequential Organ Failure Assessment, *SD* standard deviation, *D1* first day postoperative, D2 second day postoperative

### Meta-analysis

Our searches identified 1528 records. After screening based on titles and abstracts, eight articles remained for full-text assessment (Additional file 1: Figure S2). All of them met the inclusion criteria and were included in the systematic review [4, 9–15]. Details of the key characteristics of the trials are presented in Additional file 1: Table S1. Seven RCTs included more than 100 patients [4, 9, 12, 15] and one trial was multicenter [9]. The clinical settings were cardiac surgery in four trials [10, 11, 13, 15], general surgery in three studies [4, 9, 12] and hepatic surgery in one trial. The duration of the GDHT varied from 4 to 12 h. Fluids and inotropic support were used within the GDHT protocol in all included studies, while some of the RCTs also used RBC transfusion and vasodilators. Most included trials were categorized as at low risk of bias (Additional file 1: Table S2 and Figure S3).

**Fig. 3:**
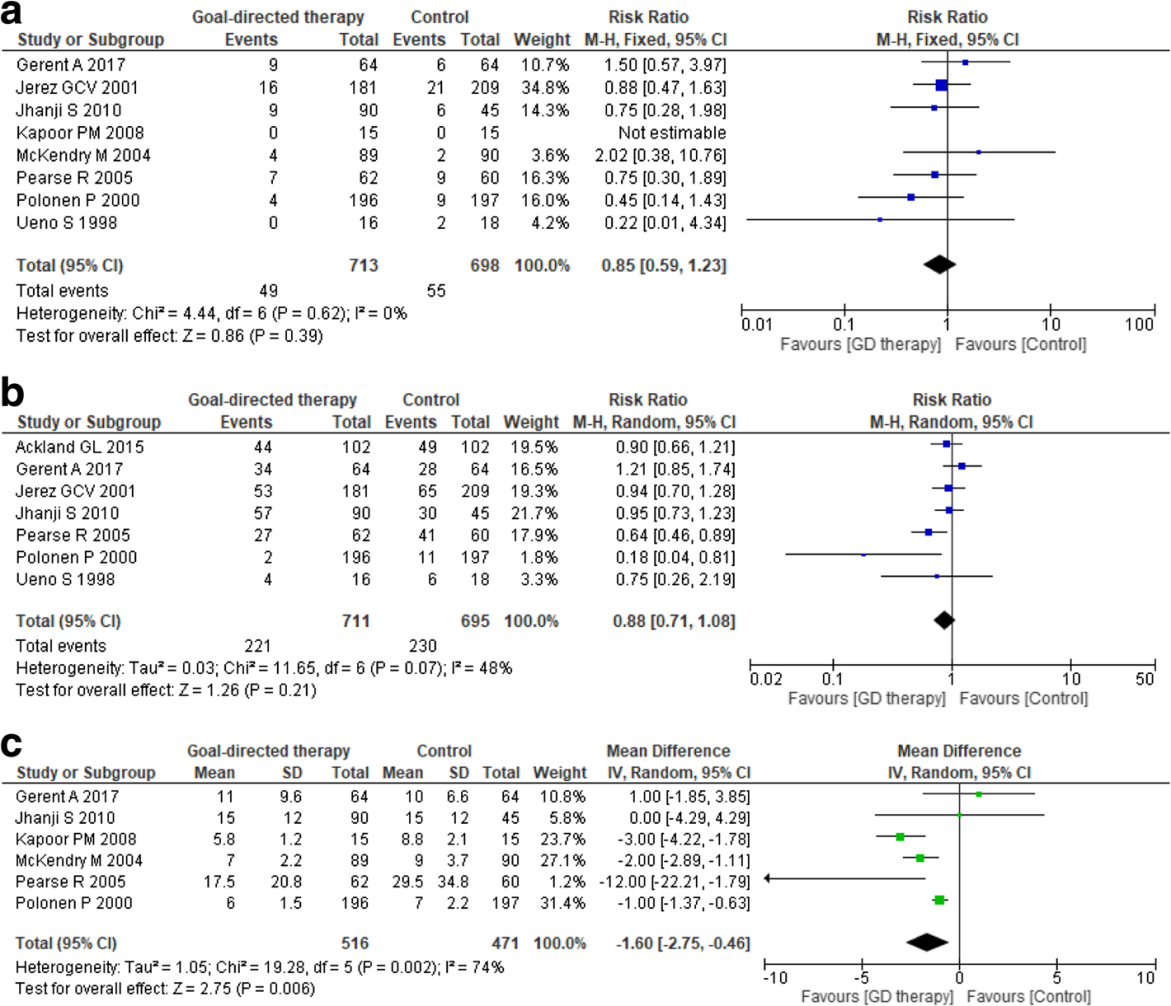
Forest plot of **a** longest follow-up mortality, **b** overall complication rate and **c** hospital length of stay. *M-H* Mantel–Haenszel method, *CI* confidence interval, *GD* goal-directed, *IV* inverse variance method

There were no differences in the longest follow-up mortality between groups (RR 0.85, 95% CI 0.59–1.23; *p* for effect = 0.4; *p* for heterogeneity = 0.7; *I*^2^ = 0%; eight included trials; Fig. 3a) and overall complication rate (RR 0.88, 95% CI 0.71–1.08; *p* for effect = 0.2; *p* for heterogeneity = 0.07; *I*^2^ = 48%; seven included trials; Fig. 3b). The lack of a GDHT effect on the overall complication rate was confirmed when sequentially removing included trials (Additional file 1: Table S3). GDHT reduced hospital LOS (MD – 1.6; 95% CI – 2.75 to − 0.46; *p* for effect = 0.006; *p* for heterogeneity = 0.002; *I*^2^ = 74%; six included trials; Fig. 3c).

## Discussion

The main finding of this RCT is that among cancer patients undergoing high-risk surgery, the use of CI-guided hemodynamic therapy applied in the first 8 postoperative hours is not associated with a reduction in 30-day mortality and severe postoperative complications when compared with protocolized hemodynamic therapy incorporating measures of perfusion and intraoperative PPV. The GDHT resulted in a higher exposure of the patients to dobutamine, without improving outcomes. After including the results of this trial in an updated systematic review and meta-analysis, we confirmed that the strategy of postoperative GDHT does not reduce overall complications and mortality in high-risk surgical patients. However, the meta-analysis suggested that postoperative GHDT is associated with a lower length of hospital stay.

GDHT is recommended in the context of enhanced recovery programs in high-risk patients [16]. A recent published meta-analysis including 45 RCTs suggested that the use of GDHT in major abdominal surgery reduces mortality and overall complications [6]. Our meta-analysis differs from this previous one because we included RCTs with a GDTH protocol performed exclusively after surgery, in the ICU. Most studies included in the meta-analysis by Sun et al. [6] start the protocol during surgery and extend it to the first 8 h of the postoperative period.

Interestingly, the best timing for performing GDHT is not specified in previous systematic reviews and recommendations. The majority of studies defined the interventions as “perioperative”, including the surgical room period and the first 6–12 h of postoperative care. We therefore speculate that most of the benefits of the GDHT protocol, if any, are the result of intraoperative protocols. Our study demonstrates that a GDHT postoperative protocol does not add benefits in outcomes when compared to a usual care protocol and might be associated with over therapy without improving outcomes.

The updated meta-analysis including nine randomized studies confirmed no reduction in mortality and overall complications while suggesting that the benefits of postoperative GDHT might be limited to a reduction in the length of hospital stay. This brings to the discussion whether it is necessary to apply postoperative GDHT in high-risk patients already undergoing an intraoperative protocol of cardiac output-guided hemodynamic algorithm as we did in our patients.

Our study differs from previous ones performed in noncardiac surgery [3–5, 7]. First, the usual care group received multimodal hemodynamic care based on the optimization of hemodynamic parameters and tissue perfusion markers, which may be enough for adequate oxygen delivery during the postoperative period. Second, during anesthesia, patients were monitored with a central venous line and an arterial line, and hemodynamic variables such as PPV, ScvO_2_ and arterial lactate were evaluated in protocolized care. Thus, it is possible that occult tissue hypoperfusion and hypoxia were avoided in the subsequent 8 h of the intervention. This is confirmed by the observation that the majority of patients were admitted to the ICU with CI > 2.5 L/min/m^2^.

In septic shock patients, Rivers et al. [17] demonstrated that goal-directed therapy aiming at adequate ScvO_2_ reduced mortality. In the last years, with advances in the process of care, more recent studies have not confirmed the benefits of applying GDHT in the ICU in septic patients [18].

The largest study evaluating the impact of cardiac output-guided hemodynamic therapy in surgical patients, the OPTIMISE trial, included 734 patients undergoing major abdominal surgery in 17 different centers. This study did not show any benefits in the intervention group for postoperative complications and mortality reduction over standard therapy [7].

In our GDHT group, patients needed more dobutamine, but we did not observe a higher risk of arrhythmia or myocardial injury, as confirmed by previous trials [7, 19]. Our GDHT protocol of care did not result in either more prescribed fluids or red blood cells, maybe because most patients were already adequately resuscitated during the intraoperative period.

We used albumin solution in the algorithm of treatment because colloid solutions are superior when compared to crystalloids in reaching hemodynamic goals [20]. In addition, previous randomized clinical studies in critically ill patients showed the safety of albumin solutions [21, 22].

We chose to use a minimally invasive noncalibrated device to measure the cardiac index. Although this device has its accuracy reduced when compared to other calibrated devices, the data are reproducible, easy to measure and easy to interpret and it is used in both surgical rooms and ICUs. In addition, physicians and nurses were already trained. We selected a cardiac index target of 2.5 L/min/m^2^ based on a previous study that was able to demonstrate improved outcomes in high-risk patients using a similar target [12].

Our study has limitations. Due to the nature of the intervention protocol, blinding was not feasible, and to reduce potential bias, outcome assessors were not aware of the study group assignment. Our results may have limited external validity because this was a single-center trial conducted in a cancer reference hospital. In addition, we analyzed a conventional hemodynamic perioperative protocol based on “normal” values of variables such as cardiac index (CI) and systolic volume. However, we have learned that population-based “normal” values do not necessarily represent the optimal values or the personal normal values of an individual. It is known that many hemodynamic variables have marked interindividual variability and depend on other factors such as biometric factors and time of disease, and probably using fixed hemodynamic goals might have contributed to the negative results of this trial. We postulate that the best concept to be applied at the bedside in high-surgical risk patients is personalized hemodynamic management, based on adaptive multiparameter hemodynamic optimization strategies targeting individual normal values adapted to the clinical situation [23–25].

This was a pragmatic study designed to analyze the effect of GDHT in the postoperative scenario of the ICU. We aimed to assess the postoperative period separately because in usual practice, independently of intraoperative management, the hemodynamic algorithm continues to be applied routinely during the first hours in the ICU. We evaluated whether applying a fixed GDHT algorithm in the postoperative period might contribute to reduce complications or even results in harm. Our results bring into discussion the need for a more personalized approach at this point, once we have shown that there is no benefit of postoperative GDHT in cancer patients undergoing major surgeries. The updated meta-analysis confirms that postoperative GDHT does not reduce overall complications and death, but might be associated with a reduced length of hospital stay.

## Conclusions

A cardiac index-guided hemodynamic therapy applied in the first 8 postoperative hours in cancer patients undergoing high-risk surgery did not reduce 30-day mortality and the rate of postoperative complications when compared with protocolized hemodynamic therapy including measures of perfusion and intraoperative PPV. This therapy was associated with an increased use of dobutamine without improving outcomes.

## Additional file

Additional file 1: Effect of postoperative goal-directed therapy in cancer patients undergoing high-risk surgery: randomized clinical trial and meta-analysis. **Appendix 1.** Surgical, anesthetic technique and intensive care treatment. **Appendix 2.** Outcome definitions. **Appendix 3.** Data collection. **Appendix 4.** Systematic review details. **Figure S1.** Cardiac index during intervention in the GDHT group (median and interquartile range). **Figure S2.** Study flow diagram of systematic review. **Figure S3.** Risk of bias graph: review authors’ judgments about each risk of bias item presented as percentages across all included studies. **Table S1.** Characteristics of included trials. **Table S2.** Risk of bias. **Table S3.** Sensitivity analysis for overall complication rate by sequential removal of each trial. Supplemental references. (DOC 255 kb)

## Abbreviations

AKI: Acute kidney injury
ARDS: Acute respiratory distress syndrome
CI: Cardiac index
DO_2_: Oxygen delivery
GDHT: Goal-directed hemodynamic therapy
HR: Heart rate
ICU: Intensive care unit
IQR: Interquartile range
MAP: Mean arterial pressure
RBC: Red blood cell
RCT: Randomized clinical trial
RR: Risk ratio
RRT: Renal replacement therapy
ScvO_2_: Central venous oxygen saturation
SD: Standard deviation
SOFA: Sepsis-related organ failure assessment
SVI: Stroke volume index
