# Supplementary material for: Effect of postoperative goal-directed therapy in cancer patients undergoing high-risk surgery: a randomized clinical trial and meta-analysis
Source: Crit Care. 2018 May 23;22:133. doi: 10.1186/s13054-018-2055-4 (PMC5964647; doi:10.1186/s13054-018-2055-4)
Supplement: Supplementary file 1 — Effect of postoperative goal-directed therapy in cancer patients undergoing high-risk surgery: randomized clinical trial and meta-analysis. Appendix 1. Surgical, anesthetic technique and intensive care treatment. Appendix 2. Outcome definitions. Appendix 3. Data collection. Appendix 4. Systematic review details. Figure S1. Cardiac index during intervention in the GDHT group (median and interquartile range). Figure S2. Study flow diagram of systematic review. Figure S3. Risk of bias graph: review authors’ judgments about each risk of bias item presented as percentages across all included studies. Table S1. Characteristics of included trials. Table S2. Risk of bias. Table S3. Sensitivity analysis for overall complication rate by sequential removal of each trial. Supplemental references. (DOC 255 kb) [file 13054_2018_2055_MOESM1_ESM.doc]

**ADDITIONAL FILE 1**

**Effect of a Postoperative Goal-Directed Therapy in Cancer Patients Undergoing High-Risk Surgery: A randomized clinical trial and systematic review**

Aline Rejane Muller Gerent, Juliano Pinheiro de Almeida, Evgeny Fominskiy, Giovanni Landoni, Gisele Queiroz de Oliveira, Stephanie Itala Rizk, Julia Tizue Fukushima, Claudia Marques Simoes, Ulysses Ribeiro Jr, Clarice Lee Park, Rosana Ely Nakamura, Rafael Alves Franco, Patricia Ines Candido, Cintia Rosa Tavares, Ligia Camara, Graziela dos Santos Rocha Ferreira, Elisangela Pinto Marinho de Almeida, Roberto Kalil Filho, Filomena Regina Barbosa Gomes Galas, Ludhmila Abrahão Hajjar

| Appendix 1. Surgical, Anesthetic technique and intensive care treatment | page 3 |
| --- | --- |
| Appendix 2. Outcome definitions | page 5 |
| Appendix 3. Data collection | page 7 |
| Appendix 4. Systematic review details | page 8 |
| Figure S1 | page 11 |
| Figure S2 | page 12 |
| Figure S3 | page 13 |
| Table S1 | page 14 |
| Table S2 | page 18 |
| Table S3 | page 26 |
| Supplemental references | page 28 |

**Appendix 1. Surgical and anesthetic technique**

Anesthesia was induced with fentanyl (3-5 μg/kg) or sufentanil (0.3-0.5 μg/kg), midazolam (0.03-0.05 mg/kg), etomidate (0.2-0.3 mg/kg) or propofol (2 mg/kg) and cisatracurium (0.2 mg/kg). Anesthesia was maintained either with inhalational agents (isoflurane or sevoflurane) in oxygen with opioids as needed or intravenously with propofol. Regional anesthesia (an epidural or subarachnoid anesthesia) was performed before surgery to provide effective analgesia of the patient’s wound. Intraoperative loading with a local anesthetic (bupivacaine or ropivacaine) and morphine or with an infusion of local anesthetic and an opioid was performed according to institutional protocols.

After tracheal intubation, all patients received invasive mechanical ventilation with intermittent positive pressure with a tidal volume of 6-8 mL/kg, positive end-expiratory pressure of 5 to 8 cm H_2_O, and fraction of inspired oxygen (FiO_2_) of 0.4 to 0.6 to maintain arterial oxygen saturation above 95%.

During surgery, all patients were monitored with a central venous line and indwelling radial artery catheter. Fluid management and administration of vasopressor and inotropic agents were performed to maintain a mean arterial pressure of 65 mmHg or higher, urinary output higher than 0.5 mL/kg/h, oxygen venous saturation equal to or greater than 70% and lactate levels lower than 2 mmol/L. Preload was optimized by fluid loading until pressure pulse variation PPV was <10%.

**Intensive care treatment**

Perioperative care was provided to both groups according to the institutional protocol, which is conforming to Enhanced Recovery After Surgery (ERAS) principles[1]. Patients were weaned from the ventilator as soon as they were hemodynamically stable with no major bleeding, normothermic, and adequate levels of consciousness and pain control achieved. Postoperative pain relief was provided to all patients. On a daily basis, we used protocols to minimize sedation and to optimize pain control, through patient controlled analgesia and epidural analgesia and also avoiding systemic opioid agents. During hospitalization, patients received antibiotic prophylaxis and thromboprophylaxis according to the procedure and clinical characteristics of patients.

Aside the study hemodynamic protocols, the postoperative established protocols included perioperative hemodynamic therapy and fluid management aiming to avoid hypervolemia with preference for balanced crystalloids and albumin. Also, perfusion tissue markers were measured at intervals of 2 hours (lactate < 3 mmol/L, ScvO_2_ > 65%) to evaluate the hemodynamic response to the interventions.

Whenever necessary, nitroglycerin and/or sodium nitroprusside were used as vasodilator; dobutamine or milrinone and/or epinephrine as inotropes; and norepinephrine and/or vasopressin as vasopressors. Weaning from the catecholamine infusion was guided by standard hemodynamic criteria. The transfusion policy was restrictive and one unit of red blood cell was administered if hemoglobin concentration was lower than 8 g/dL, with exceptions according to hemodynamic status and cardiovascular decompensation.

We started early oral fluid intake whenever possible. If patients were not able to intake, parenteral fluids were given in a rate of 40 mL/h of Ringer lactate solution and fluid losses replacement. We kept all patients under protective ventilation strategy, with these goals: low tidal volume (VT < 8 mL/kg); driving pressure < 15 cmH_2_O; non-invasive ventilation and oxygen supplementation (SpO_2_ > 92%) aiming early weaning of mechanical ventilation.

The nutritional support was based on early enteral feeding, with clear fluids given at least 2 hours after surgery and regular diet beginning postoperative day 1 pending on clinical judgment.

A glycemic control protocol was used regularly keeping glucose levels < 180 mg/dL. We applied in the ICU a daily round checklist focusing in early removal of devices, glycemic control goals, deep venous prophylaxis, stress ulcer prophylaxis and early mobilization.

Decision to transfer the patient from the ICU to the ward was based on the following criteria: absence of acute organ failure, adequate cardiac and respiratory stability with no hemodynamically significant arrhythmias, no intravenous inotropic or vasopressor agent, and no seizure activity. Criteria for hospital discharge were hemodynamic and cardiac rhythm stability, presence of clean and dry incisions, afebrile, normal bowel movement, and independent ambulation and feeding.

**Appendix 2. Outcomes definition**

The primary outcome was a composite endpoint of 30-day mortality and major clinical complications during hospital stay. Clinical complications were defined as acute kidney injury, stroke, myocardial infarction, acute decompensated heart failure, pulmonary thromboembolism, mesenteric ischemia, peripheral vascular ischemia, acute respiratory distress syndrome, deep wound infection and reoperation. The components of the primary outcome were defined as follows:

- 30-day mortality was defined as death during hospital stay or death after hospital discharge until 30 days following the procedure;
- Stroke was defined by a new focal deficit lasting longer than 24 hours and associated with a compatible brain computed tomography imaging;
- Reoperation due to surgical complications;
- ARDS was defined by the Berlin definition [2];
- Mesenteric arterial ischemia was defined as an occlusive or non-occlusive impairment of intestinal blood flow leading to bowel ischemia diagnosed by arteriography or surgery;
- Pulmonary thromboembolism refers to obstruction of the pulmonary artery or one of its branches by thrombus that originated elsewhere in the body;
- Deep wound infection was defined as infection that involves deep tissues, such as fascial and muscle layers. This also included infection involving both superficial and deep incision sites;
- Acute decompensated heart failure was defined the development of acute dyspnea associated with the rapid accumulation of fluid within the lungs interstitial and alveolar spaces as a result of elevated cardiac filling pressures;
- Acute kidney injury (AKI): defined by the Acute Kidney Injury Network classification. The criteria for AKI diagnosis are: an abrupt (within 72 hours) reduction in kidney function defined as an absolute increase in serum creatinine of 0.3 mg/dL and more, or a percentage increase in serum creatinine of 50% or more (1.5-fold from baseline), or a reduction in urine output (documented oliguria of < 0.5 mL/kg/h for >6 h) [3]. Renal function was evaluated daily and AKI was defined by an AKIN stage 1 or higher. The need for renal replacement therapy (RRT) was based on both clinical and laboratory assessment [4];
- Myocardial ischemia was defined by elevation of myocardial enzymes (creatine kinase MB level at least 5 times the normal upper limit or troponin I values greater than 5 ng/mL) during the first 48 hours, associated with one of the following: new pathological Q waves, coronary artery occlusion documented by angiography, or imaging evidence of new loss of viable myocardium [5];
- Lower limb ischemia was defined by an abrupt reduction of the arterial perfusion of the limb characterized by the absence of arterial pulse, pallor, cyanosis or ischemic skin lesions and a duplex ultrasound showing absence of arterial blood flow;
- Septic shock was defined by standard criteria[6];
- Length of ICU was defined as the median duration of days of ICU stay;
- Length of hospital stay was defined as the median duration of days since admission until hospital discharge;
- 90-day mortality was defined as death during hospital stay or death after hospital discharge until 90 days following the procedure. A phone call was performed to check the status vital of patients.

**Appendix 3. Data collection**

After randomization, we recorded demographic, hemodynamic and clinical data. We collected data on preoperative comorbidity, intraoperative and postoperative management and clinical outcomes. Organ failure was assessed using the Sequential Organ Failure Assessment (SOFA) [7] score, calculated using the worst values within the first 24 and 48 hours after ICU admission. Preoperative renal function was calculated based on the serum creatinine dosage the day before surgery. The values of two scores used to assess the degree of functional impairment, the Karnofsky Performance Status scale (from 0 [most impaired] to 100 [least impaired]) and the Eastern Cooperative Oncology Group scale (from 0 [most impaired] to 5 [least impaired]), were also recorded [8, 9].

We also evaluated the Charlson comorbidity index, a score used to predict outcome according to the weighted presence of various comorbid conditions, which can also be adjusted by patient age (higher score, higher risk of death) [10].

We also collected data related to characteristics of the surgical procedure including type of surgery, type and amount of fluid (including blood transfusions), and laboratory data during the intraoperative period, such as hemoglobin levels and hematocrit, central venous oxygen saturation (ScvO_2_) and lactate concentration at baseline (just after induction of anesthesia) and at the end of the procedure.

During the ICU stay, all patients were assessed daily by investigators and clinical and laboratory data were recorded. The following hemodynamic parameters were assessed: mean arterial pressure (MAP), heart rate (HR), cardiac index (CI), at ICU admission (D0), and after ICU admission at 2nd hour (T2), and 8th hour (T8) were measured. Markers of tissue hypoxia as lactate, base excess (BE), mixed venous saturation (ScvO_2_), and gap of CO_2_ on the same timepoints were evaluated. Hemoglobin, hematocrit, serum creatinine and biomarkers such as creatine-kinase MB (CK-MB) and troponin I, were collected daily until the ICU discharge.

During 30 days, we evaluated the incidence of mortality or severe complications including acute kidney injury, stroke, myocardial infarction, acute decompensated heart failure, pulmonary thromboembolism, mesenteric ischemia, peripheral vascular ischemia, acute respiratory distress syndrome, deep wound infection and reoperation. We also evaluated the incidence of septic shock, development of AKI requiring renal replacement therapy (RRT), ICU readmission rate, ICU and hospital lengths of stay (LOS), 7-day SOFA score and 90-day mortality.

After discharge from the ICU, clinical outcomes were evaluated on the regular ward. The clinical follow-up after hospital discharge was performed by telephone at the 90th postoperative day. The outcome and safety data were collected in appropriate case report forms.

**Appendix 4. Systematic Review and Meta-analysis details**

Detailed methods

We performed the systematic review and meta-analysis in accordance with Preferred Reporting Items for Systematic Reviews and Meta-Analysis (PRISMA) statement. Two authors independently searched PubMed/Medline, Embase, Scopus, Cochrane Central Register of Controlled Trials databases, and Google Scholar for RCTs investigating postoperative GDT use in high-risk surgery patients published before May 1st, 2017. The computer-based searches combined terms related to GDT (eg, cardiac output-guided hemodynamic therapy), and randomized evidence (eg, randomized controlled trial) in humans, without any language restriction. Detailed PubMed search strategy is: (Surgery[tiab]) AND (((((((Hemodynamics[tiab] OR "Cardiac Output"[Mesh]) OR "Stroke Volume"[Mesh]) OR (("oxygen"[MeSH Terms] OR "oxygen"[All Fields]) AND ("delivery, obstetric"[MeSH Terms] OR ("delivery"[All Fields] AND "obstetric"[All Fields]) OR "obstetric delivery"[All Fields] OR "delivery"[All Fields]))) OR "oxygen delivery"[All Fields]) OR (early[All Fields] AND ("goals"[MeSH Terms] OR "goals"[All Fields] OR "goal"[All Fields]) AND directed[All Fields] AND ("therapy"[Subheading] OR "therapy"[All Fields] OR "therapeutics"[MeSH Terms] OR "therapeutics"[All Fields]))) OR "early goal directed therapy"[All Fields]) OR "Dobutamine"[Mesh]) OR ("dopexamine"[Supplementary Concept] OR "dopexamine"[All Fields])) AND "humans"[MeSH Terms] AND Randomized Controlled Trial[ptyp] AND English[lang] AND "adult"[MeSH Terms])

Two researches independently screened the titles and abstracts of all initially identified studies according to the selection criteria. Full-text articles of studies that met all selection criteria were retrieved. Reference lists of the selected studies, relevant reviews and meta-analyses identified on the topic were manually searched for additional articles.

Inclusion criteria were: (1) studies of adult high-risk patients (author definition) undergoing cardiac and non-cardiac surgery, (2) postoperative goal-directed therapy versus conventional postoperative treatment, (3) presenting postoperative complications and mortality data, (4) randomized controlled trials. Trials comparing GDT versus usual care during only intraoperative period, trials comparing GDT versus usual care during perioperative period, studies published as a meeting abstract, or non-randomized studies were excluded.

Two authors independently extracted data and a consensus was reached in case of any inconsistency with involvement of a third author. The data extracted included first author, year of publication, journal, sample size, clinical settings, mortality data, timing of GDT therapy initiation, complications and termination.

Two authors assessed the risk of bias using the Cochrane Collaboration’s Risk of Bias tool. Due to the nature of the studied intervention, blinding of participants and personnel was not possible in all included trials. Therefore, we did not consider this criterion in the final conclusion as a potential risk of bias. We therefore determined the risk of bias of the included RCTs using the following criteria: random sequence generation, allocation concealment, blinding of outcome assessment, incomplete outcome data, selective reporting, and other bias.

The primary outcome was the longest follow-up all-cause mortality. Summary measure was presented as risk ratio (RR) with 95% confidence interval (CI). The Mantel-Haenszel method was used to combine summary measures using random effects model to minimize the effect of between-study heterogeneity. We evaluated heterogeneity between studies using Cochran’s Q (represented as χ² and p values) and the I² statistic, which describes the percentage of variation between studies that is due to heterogeneity. In accordance with Cochrane guidance, we did not analyse publication bias because our search identified fewer than ten studies for each data comparison. We performed sensitivity analyses by sequential removing of trials. Data from each trial were considered as per the intention-to-treat principle. All statistical tests were two-sided and used a significance level of p < 0.05. Review Manager (RevMan, Version 5.3., Copenhagen: The Nordic Cochrane Centre, The Cochrane Collaboration, 2014) and STATA (Version 11; StataCorp, College Station, TX) were used for all statistical analyses.

Figure S1. Cardiac index during intervention in the GDHT group (median and interquartile range).

Figure S2. Study flow diagram of the systematic review.


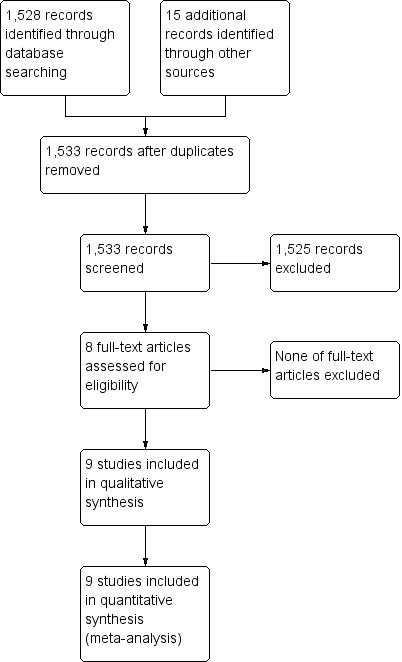


Figure S3. Risk of bias graph: review authors' judgements about each risk of bias item presented as percentages across all included studies.


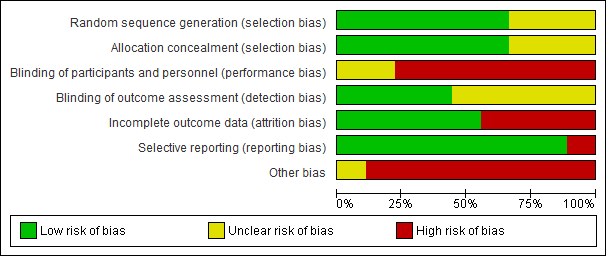


Table S1. Characteristics of the included trials.

| Author and year | Journal | Participants | Intervention | Timing | Goals of therapy | Devise | Control group |
| --- | --- | --- | --- | --- | --- | --- | --- |
| Ackland GL 2015 | Lancet Respir Med | Major elective surgery with duration ≥120 min in high-risk patients: ASA ≥3, risk of postoperative morbidity >50%, modified Revised Cardiac Score ≥3, age ≥70 years, history of CVD, cardiac failure, poor exercise capacity, renal impairment, diabetes | Fluids, inotropes | 6 hours after surgery | Preoperative oxygen delivery value for each individual patient by maximizing stroke volume and ensuring HR not more than 25% above baseline or <100 beats min^-1^, SpO_2_ ≥94%, Hb >80 g L^-1^, core temperature ≥36ºC, MAP 60-90 mmHg | LiDCOPlus cardiac output monitor | SpO_2_ ≥94%, Hb >80 g L^-1^, cor temperature ≥36ºC, HR <100 beats min^-1^, MAP 60-90 mmHg |
| Gerent A 2017 | Submitting | Adult oncologic patients undergoing high-risk non-cardiac surgery | Fluids, inotropes, and blood transfusions | 8 hours after surgery | CI ≥2.5 L min^-1^ m^-2^, SaO_2_ >95%, Hb >80 g L^-1^, SvO_2_ >70%, MAP >70 mmHg, HR <100 beats min^-1^, lactate <3 mmol L^-1^ | FloTrac-Vigileo™ cardiac output monitor | SaO_2_ >95%, Hb >80 g L^-1^, SvO_2_ >70%, MAP >70 mmHg, HR <100 beats min^-1^, lactate <3 mmol L^-1^ |
| Jerez GCV 2001 | Medicina Intensiva | Heart surgery with CPB | Fluids, inotropes | 8 hours after surgery | SvO_2_ ≥70% | Thermodilution pulmonary arterial catheter | Standard hospital treatment |
| Jhanji S 2010 | Crit Care | Major elective gastrointestinal surgery | Fluids, inotropes | 8 hours after surgery | Sustained 10% rise in SV for ≥20 min, HR <100 beats min^-1^ or increase <20% above baseline, MAP 60-100 mmHg, CVP 6-12 mmHg, UO >25 mL h^-1^, Hb > 8 g dL^-1^, SpO_2_ >94%, temperature 36-37ºC, BE -2 to 2 mmol L^-1^, PaCO_2_ 35-45 mmHg | LiDCOPlus cardiac output monitor | Sustained 2 mmHg rise in CVP for ≥20 min, HR 60-100 beats min^-1^, MAP 60-100 mmHg, CVP 6-12 mmHg, UO >25 mL h^-1^, Hb > 8 g dL^-1^, SpO_2_ >94%, temperature 36-37ºC, BE -2 to 2 mmol L^-1^, PaCO_2_ 35-45 mmHg |
| Kapoor PM 2008 | Ann Card Anaesth | Coronary artery bypass surgery on CPB with an EuroSCORE ≥3 | Fluids, inotropes, vasodilators, and blood transfusions | 8 hours after surgery | CI 2.5-4.2 l min^-1^ m^-2^, SVI 30-65 mL beat^-1^ m^-2^, SVRI 1500-2500 dyne s cm^-5^ m^-2^, DO_2_I 450-600 ml min^-1^ m^-2^, ScvO_2_ >70%, SVV <10%, CVP 6-8 mmHg, MAP 90-105 mmHg, SpO_2_ >95%, pH 7.35-45, PaO_2_ >100 mmHg, PaCO_2_ 35-45 mm Hg, Ht >30%, UO >1 mL kg^-1^ h^-1^ | FloTrac™ cardiac output monitor, PreSep™ catheter for continuous central venous oximetry | CVP 6-8 mmHg, MAP 90-105 mmHg, Ht >30%, arterial blood gases and UO were monitored hourly |
| McKendry M 2004 | BMJ | Cardiac surgery on CPB | Fluids, inotropes, and vasodilators | 4 hours after surgery | SVI ≥35 mL min^-1^ m^-2^ | Oesophageal Doppler | Arterial and central venous pressures, urine output, arterial base deficit |
| Pearse R 2005 | Crit Care | High-risk general surgical patients | Fluids, inotropes | 8 hours after surgery | Sustained 10% rise in SV for ≥20 min, SpO_2_ >94%, Hb >8 g dL^-1^, temperature 37ºC, HR <100 beats min^-1^ or <20% above baseline, MAP 60-100 mmHg, DO_2_I >600 mL min^-1^ m^-2^, UO >5 mL kg^-1^ h^-1^, 2-hours lactate rise <2 mmol L^-1^, CI >2.5 L min^-1^ m^-2^ | LiDCOPlus cardiac output monitor | Sustained 2 mmHg rise in CVP for ≥20 min, SpO_2_ >94%, Hb >8 g dL^-1^, temperature 37ºC, HR <100 beats min^-1^ or <20% above baseline, MAP 60-100 mmHg, UO >5 mL kg^-1^ h^-1^, 2-hours lactate rise <2 mmol L^-1^, CI >2.5 L min^-1^ m^-2^ |
| Polonen P 2000 | Anesth Analg | Elective cardiac surgery | Fluids, inotropes, vasodilators, and blood transfusions | 8 hours after surgery | SvO_2_ >70%, lactate ≤2.0 mmol L^-1^, CI >2.5 L min^-1^ m^-2^, PCWP 12-18 mmHg, MAP 60-90 mmHg, Hb ≥100 g L^-1^ | Thermodilution pulmonary arterial catheter | CI >2.5 L min^-1^ m^-2^, PCWP 12-18 mmHg, MAP 60-90 mmHg, Hb ≥100 g L^-1^ |
| Ueno S 2008 | Surgery | Elective hepatectomy for hepatocellular carcinoma and had biopsy-proven advanced chronic active hepatitis or liver cirrhosis | Fluids, inotropes | 12 hours after surgery | CI >4.5 L min^-1^ m^-2^, DO_2_I >600 mL min^-1^ m^-2^, VO_2_ >170 mL min^-1^ m^-2^, AP 100/60 mmHg, CVP <15 mmHg, Hb >10 g dL^-1^, UO >30 mL h^-1^, HR 60-120 beats min^-1^, temperature <38ºC, PaO_2_ >90 mmHg, pH 7.3-7.5, PvO_2_ >38 mmHg, mean PA pressure <19 mmHg, PCWP 9-18 mmHg | Oximetrix 3 SO_2_/CO Computer thermodilution cardiac output monitor, Opticath model P7110 fiberoptic catheter | CI 2.8-4.0 L min^-1^ m^-2^, AP >100/60 mmHg, CVP 4-12 mmHg, Hb >10 g dL^-1^, UO >30 mL h^-1^, HR 60-120 beats min^-1^, temperature <38ºC, PaO_2_ >90 mmHg, pH 7.3-7.5, PvO_2_ >38 mmHg, mean PA pressure 11-15 mmHg, PCWP 4-12 mmHg |

AP, arterial pressure; ASA, American Society of Anesthesiologists physical status classification system; BE, base excess; CI, cardiac index; CPB, cardio-pulmonary bypass; CVD, cardio-vascular disease; CVP, central venous pressure; DO_2_I, delivery oxygen index; HR; heart rate; Ht, haematocrit; MAP, mean arterial pressure; PA, pulmonary artery; PaO_2_, partial oxygen pressure in arterial blood; PaCO_2_, partial carbon dioxide pressure in arterial blood; PCWP, pulmonary capillary wedge pressure; PvO_2_, partial oxygen pressure in venous blood; ScvO_2_, central venous saturation of oxygen; SpO_2_, saturation of oxygen measured by pulse oximetry; SV, stroke volume; SVI, stroke volume index; SvO_2_, mixed venous saturation of oxygen; SVRI, systemic vascular resistance index; SVV, stroke volume variation; UO, urine output; VO_2_, oxygen consumption

Table S2. Risk of bias.

| Study | Bias | Author’s judgement | Support for judgement |
| --- | --- | --- | --- |
| Ackland GL 2015[3] | Random sequence generation | Low | “The randomisation list was generated by Stata, stratified by hospital…” |
|  | Allocation concealment | Low | “…then concealed by envelope. Participants were centrally allocated to treatment groups.” |
|  | Blinding of participants and personnel | High | “the medical research and nursing staff delivering the haemodynamic protocol did not reveal the study group allocation to critical care staff, attending surgical, or physician teams.” |
|  | Blinding of outcome assessment | Low | “Morbidity outcomes were verified… by an investigator masked to the intervention. Grading of severity was verified at the end of the trial by an independent assessor masked to the intervention.” |
|  | Incomplete outcome data | High | 7 patients in the treatment group and 10 patients in the control group were excluded due to adverse intraoperative events or substantial changes in planned surgery because of disseminated malignancy |
|  | Selective reporting | High | Primary outcome was defined as “Reduction in post-operative morbidity on day 3 post-operatively as defined by the Post-Operative Morbidity Survey” according to the trial registration ISRCTN76894700. In the manuscript the authors used the Clavien-Dindo scale for estimation postoperative morbidity. There are differences between inclusion criteria between the registered protocol and the manuscript. The protocol was registered retrospectively |
|  | Other bias | High | For sample size calculation the level of 15% of patients was taken into account when the authors considered that some patients in the control group are able to achieve the oxygen delivery target spontaneously. But the level of postoperative oxygen delivery achievers in the control group was substantially higher than the authors had anticipated |
| Gómez-Coronado VJ 2001 [12] | Random sequence generation | Unclear | Not stated |
|  | Allocation concealment | Unclear | Not stated |
|  | Blinding of participants and personnel | Unclear | Not stated |
|  | Blinding of outcome assessment | Unclear | Not stated |
|  | Incomplete outcome data | Low | All patients were included in the analysis |
|  | Selective reporting | Low | Apparently free of selecting reporting |
|  | Other bias | High | There is no information about sample size calculation |
| Jhanji S 2010 [13] | Random sequence generation | Low | “…computer-generated random sequence in blocks of nine. Groups were stratified according to surgical procedure…” |
|  | Allocation concealment | Low | “Study group allocations were placed in serially numbered opaque envelopes” |
|  | Blinding of participants and personnel | High | “Only the member of the research team who delivered the intervention was aware of the study group allocation” |
|  | Blinding of outcome assessment | Low | “Clinical outcomes data for each patient were collected by a member of the research team who was unaware of study group allocation and then verified by the senior investigator who was also unaware of the study group allocation” |
|  | Incomplete outcome data | Low | All patients were included in the analysis |
|  | Selective reporting | Low | Apparently free of selecting reporting |
|  | Other bias | High | According to the trial registration ISRCTN94850719 at the beginning the primary outcome was determined as reduction in post-operative complication rates. Also at the beginning two groups were planned. 23.10.2007 the authors made amendments in the protocol by changing the primary outcome, adding the third treatment group, changing the planned number of the patients. In the manuscript the authors investigated microvascular flow and systemic markers of inflammation that were not indicated in the registered protocol. Cardiac output monitors were provided on loan by LiDCO Ltd. One author received a research grant and equipment loans from LiDCO Ltd and honoraria from Edwards Lifescience and Pulsion Medical Systems. Three authors were named inventors on a lapsed patent application relating to the perioperative use of dopexamine |
| Kapoor PM 2008 [14] | Random sequence generation | Unclear | Not stated |
|  | Allocation concealment | Low | “… the sealed envelope technique” |
|  | Blinding of participants and personnel | High | “… the study could not be blinded” |
|  | Blinding of outcome assessment | Unclear | Not stated |
|  | Incomplete outcome data | High | Two patients in the treatment group were excluded because of intra-aortic balloon pump use and postoperative atrial fibrillation. One patients in the control group was excluded because of intra-aortic balloon pump use |
|  | Selective reporting | Low | Apparently free of selecting reporting |
|  | Other bias | High | Small sample size. There is no calculation for sample size. There are several outcomes, without determination one of them as a primary. Correction for multiple comparison was not performed |
| McKendry M 2004 [15] | Random sequence generation | Low | “…priori computer generated sequence” |
|  | Allocation concealment | Unclear | “The study nurse opened the serially numbered, sealed, opaque envelopes on arrival of patients at intensive care” |
|  | Blinding of participants and personnel | High | The study nurse took readings from the patients with an oesophageal Doppler probe” |
|  | Blinding of outcome assessment | Unclear | Non stated |
|  | Incomplete outcome data | High | Five patients in the control group were excluded from the analysis due to they did not receive intervention because were too unstable |
|  | Selective reporting | Low | Apparently free of selecting reporting |
|  | Other bias | High | At 4 hours after randomization 35 patients (39%) in the treatment group had not predefined values of stroke volume index. Deltex provided research funds to the department and contributed towards the salaries of the research nurses and travel expenses and registration fees to enable them to present their research at national and international congresses. One author did consultancy work for Deltex. |
| Pearse R 2005 [16] | Random sequence generation | Low | “Patients were assigned … by computer-generated random sequence” |
|  | Allocation concealment | Low | “Study group assignments were placed in serially numbered opaque envelopes” |
|  | Blinding of participants and personnel | High | The study treatments were administered by a member of the research team who was the only individual aware of study group allocation |
|  | Blinding of outcome assessment | Low | Complications were verified, in accordance with predefined criteria, by a member of the research team unaware of study group allocation |
|  | Incomplete outcome data | Low | All patients were included in the analysis |
|  | Selective reporting | Low | Apparently free of selecting reporting |
|  | Other bias | High | The planned sample size was 300 patients. “The study was stopped early on the advice of the external safety assessor, after assessment of data from the first 100 patients, because the primary end-point had been achieved”. Thirteen patients in the treatment group (21%) did not achieve the predefined study goals. “Recruitment was possible only when a member of the research team was available to take informed consent before surgery and administer the 8-hour study protocol”. One author received a travel grant from LiDCO Ltd. One author has previously (before the study) performed consultancy work for LiDCO Ltd. One author performed (during the study) consultancy work for LiDCO Ltd |
| Polonen P 2000 [17] | Random sequence generation | Unclear | Not stated |
|  | Allocation concealment | Low | “… sealed envelope technique” |
|  | Blinding of participants and personnel | High | “… the caregivers were aware of the randomization group” |
|  | Blinding of outcome assessment | Unclear | Not stated |
|  | Incomplete outcome data | High | Nine patients were excluded from the trial. Eight patients were already enrolled in another study, including a Jehovah’s witness because the protocol algorithm required red blood cell infusion. One patient died during the operation |
|  | Selective reporting | Low | Apparently free of selecting reporting |
|  | Other bias | High | In the treatment group 84 patients (43%) did not achieve the targets at 8 hours of the protocol treatment |
| Gerent A 2017 | Random sequence generation | Low | “Randomization was performed only after patient enrolment, with a computer-generated list in a 1:1 ratio, generated on-line by a web-based program…” |
|  | Allocation concealment | Low | “The investigator opened the serially numbered, sealed, opaque envelopes on arrival of patients at intensive care…” |
|  | Blinding of participants and personnel | High | “The nature of the intervention precluded blinding of the patients and attending physicians.” |
|  | Blinding of outcome assessment | Low | “Outcome assessors were unaware of the assigned treatment.” |
|  | Incomplete outcome data | Low | All patients were included in the analysis |
|  | Selective reporting | Low | Free of selecting reporting according to the trial registration NCT01946269 |
|  | Other bias | Unclear | Apparently free of other bias |
| Ueno S 2008 [11] | Random sequence generation | Low | “… stratified blocked randomization method” |
|  | Allocation concealment | Unclear | Not stated |
|  | Blinding of participants and personnel | Unclear | Not stated |
|  | Blinding of outcome assessment | Unclear | Not stated |
|  | Incomplete outcome data | Low | All patients were included in the analysis |
|  | Selective reporting | Low | Apparently free of selecting reporting |
|  | Other bias | High | Small sample size. There is not calculation for sample size |

Table S3. Sensitivity analysis for overall complication rate by sequential removal of each trial

| Excluded trial | Risk Ratio [95% Confidence Interval] | P value |
| --- | --- | --- |
| **All trials included** | **0.88 [0.71, 1.08]** | **0.21** |
| Ackland GL 2015 | 0.86 [0.66, 1.12] | 0.26 |
| Gerent A 2017 | 0.83 [0.67, 1.02] | 0.07 |
| Jerez GCV 2001 | 0.85 [0.66, 1.11] | 0.23 |
| Jhanji S 2010 | 0.85 [0.65, 1.11] | 0.22 |
| Pearse R 2005 | 0.95 [0.79, 1.15] | 0.58 |
| Polonen P 2000 | 0.90 [0.76, 1.07] | 0.23 |
| Ueno S 1998 | 0.88 [0.70, 1.10] | 0.25 |

**Appendix 5. PubMed search strategy**

**SUPPLEMENTAL REFERENCES**

1. Ljungqvist O, Scott M, Fearon KC: Enhanced Recovery After Surgery: A Review. JAMA Surg 2017, 152:292-298.

2. Ranieri VM, Rubenfeld GD, Thompson BT, Ferguson ND, Caldwell E, Fan E, Camporota L, Slutsky AS, Force ADT: Acute respiratory distress syndrome: the Berlin Definition. JAMA 2012, 307:2526-2533.

3. Ackland GL, Iqbal S, Paredes LG, Toner A, Lyness C, Jenkins N, Bodger P, Karmali S, Whittle J, Reyes A *et al*: Individualised oxygen delivery targeted haemodynamic therapy in high-risk surgical patients: a multicentre, randomised, double-blind, controlled, mechanistic trial. Lancet Respir Med 2015, 3:33-41.

4. Haase M, Bellomo R, Matalanis G, Calzavacca P, Dragun D, Haase-Fielitz A: A comparison of the RIFLE and Acute Kidney Injury Network classifications for cardiac surgery-associated acute kidney injury: a prospective cohort study. J Thorac Cardiovasc Surg 2009, 138:1370-1376.

5. Thygesen K, Alpert JS, White HD, Infarction JEAAWTFftRoM: Universal definition of myocardial infarction. J Am Coll Cardiol 2007, 50:2173-2195.

6. Dellinger RP, Levy MM, Rhodes A, Annane D, Gerlach H, Opal SM, Sevransky JE, Sprung CL, Douglas IS, Jaeschke R *et al*: Surviving sepsis campaign: international guidelines for management of severe sepsis and septic shock: 2012. Critical care medicine 2013, 41:580-637.

7. Jones AE, Trzeciak S, Kline JA: The Sequential Organ Failure Assessment score for predicting outcome in patients with severe sepsis and evidence of hypoperfusion at the time of emergency department presentation. Critical care medicine 2009, 37:1649-1654.

8. Schag CC, Heinrich RL, Ganz PA: Karnofsky performance status revisited: reliability, validity, and guidelines. J Clin Oncol 1984, 2:187-193.

9. Oken MM, Creech RH, Tormey DC, Horton J, Davis TE, McFadden ET, Carbone PP: Toxicity and response criteria of the Eastern Cooperative Oncology Group. Am J Clin Oncol 1982, 5:649-655.

10. Charlson ME, Pompei P, Ales KL, MacKenzie CR: A new method of classifying prognostic comorbidity in longitudinal studies: development and validation. J Chronic Dis 1987, 40:373-383.

11. Ueno S, Tanabe G, Yamada H, Kusano C, Yoshidome S, Nuruki K, Yamamoto S, Aikou T: Response of patients with cirrhosis who have undergone partial hepatectomy to treatment aimed at achieving supranormal oxygen delivery and consumption. Surgery 1998, 123:278-286.

12. Gómez-Coronado VJ, Marcos MR, Civantos DP, Ruiz JT, Torres BJ, Gómez-Coronado IB, Pinna MR, Bernayaki MZ, Mata JAF: Hemodynamic optimazation and morbimortality after heart surgery. Med Intensiva 2001, 25:297-302.

13. Jhanji S, Vivian-Smith A, Lucena-Amaro S, Watson D, Hinds CJ, Pearse RM: Haemodynamic optimisation improves tissue microvascular flow and oxygenation after major surgery: a randomised controlled trial. Crit Care 2010, 14:R151.

14. Kapoor PM, Kakani M, Chowdhury U, Choudhury M, Lakshmy, Kiran U: Early goal-directed therapy in moderate to high-risk cardiac surgery patients. Ann Card Anaesth 2008, 11:27-34.

15. McKendry M, McGloin H, Saberi D, Caudwell L, Brady AR, Singer M: Randomised controlled trial assessing the impact of a nurse delivered, flow monitored protocol for optimisation of circulatory status after cardiac surgery. BMJ 2004, 329:258.

16. Pearse R, Dawson D, Fawcett J, Rhodes A, Grounds RM, Bennett ED: Early goal-directed therapy after major surgery reduces complications and duration of hospital stay. A randomised, controlled trial [ISRCTN38797445]. Crit Care 2005, 9:R687-693.

17. Polonen P, Ruokonen E, Hippelainen M, Poyhonen M, Takala J: A prospective, randomized study of goal-oriented hemodynamic therapy in cardiac surgical patients. Anesth Analg 2000, 90:1052-1059.
